# Acute lower limb malperfusion triggered by a large vegetation located on the proximal entry tear of chronic type B aortic dissection

**DOI:** 10.1016/j.jvscit.2022.06.017

**Published:** 2022-07-18

**Authors:** Takanori Tsujimoto, Masamichi Matsumori, Katsuhiro Yamanaka, Kenji Okada

**Affiliations:** Division of Cardiovascular Surgery, Department of Surgery, Kobe University Graduate School of Medicine, Kobe, Japan

**Keywords:** Lower limb malperfusion, Proximal entry tear, Transesophageal echocardiography, Type B aortic dissection, Vegetation

## Abstract

A 74-year-old man who had been receiving antibiotic treatment for meningitis was transferred to our hospital because of a sudden decrease in lower limb blood pressure. Computed tomography revealed a type B aortic dissection with obstruction of the abdominal aorta. Furthermore, transesophageal echocardiography revealed a large vegetation on the proximal entry tear of the descending aorta. We performed successful emergency descending and abdominal aorta replacement, which prevented complications from intraoperative organ malperfusion. In the present report, we have described an effective treatment for lower limb malperfusion complicated by a combination of chronic aortic dissection and bacteremia.

Lower limb malperfusion (LLM) syndrome will occur in 5% to 12% of type B aortic dissection (TBAD) cases and has been associated with high 30-day mortality.^1^ LLM syndrome usually develops during the acute phase of aortic dissection. However, in the present report, we have described an extremely rare case of severe LLM syndrome during the chronic phase of TBAD. The syndrome had been triggered by a large vegetation located on the proximal entry tear of the descending aorta. The patient had provided written informed consent for the report of his case details. The ethical committee of our institution approved the present study (approval no., B190201).

## Case report

**Fig 1:**
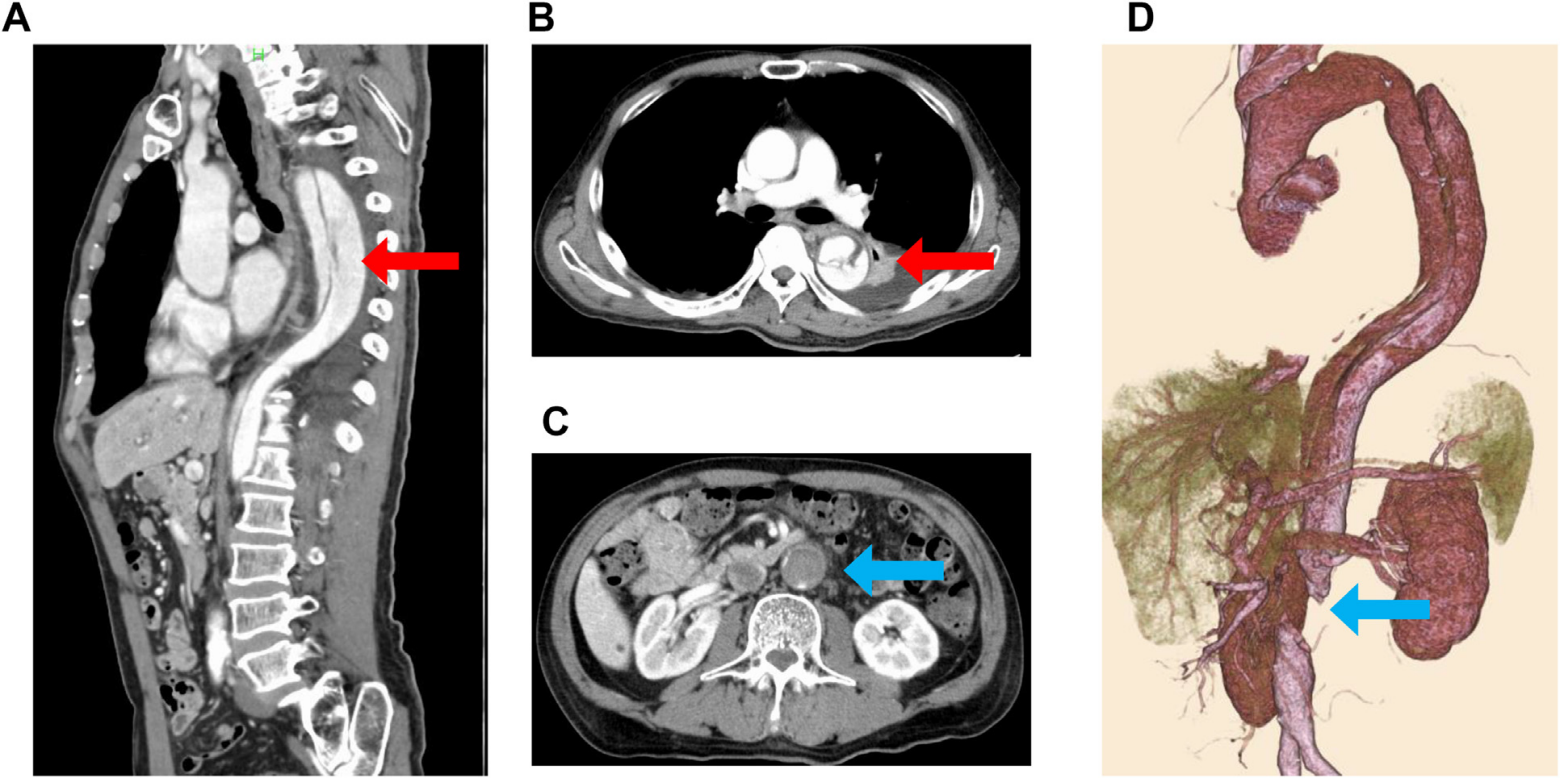
Three-dimensional reconstruction of contrast-enhanced computed tomography images in mid-sagittal view **(A)**, axial view at the proximal descending aorta level **(B)**, and axial view at infrarenal abdominal aorta level **(C)**. *Red arrows* indicate an entry tear; and *blue arrows,* an occluded region of the abdominal aorta.

**Fig 2:**
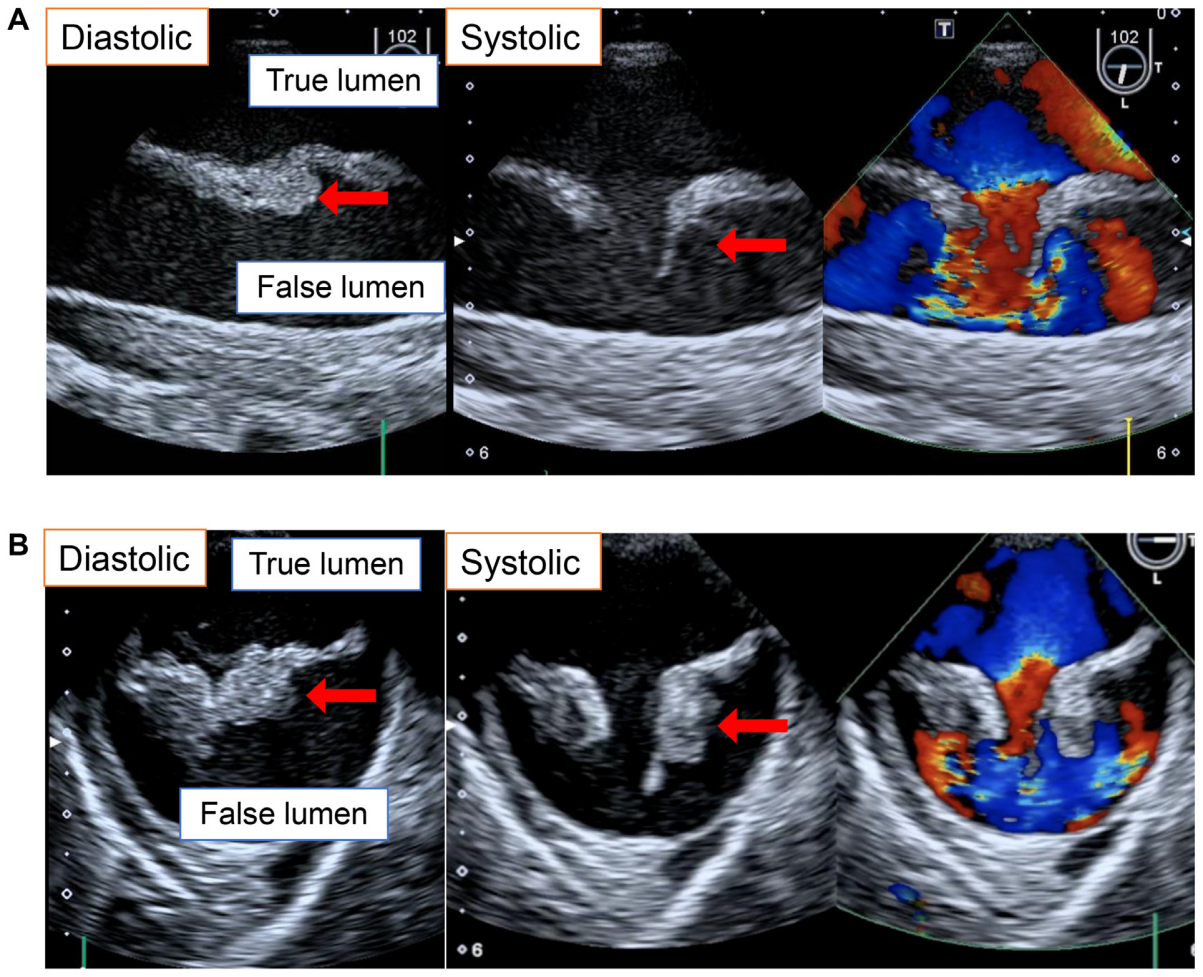
Echocardiographic images of proximal descending aorta with and without color Doppler in long-axis view **(A)** and short-axis view **(B)**.

A 74-year-old man who had presented with persistent fever and disturbance of consciousness had been admitted to a different hospital. Because gram-positive cocci (*Streptococcus agalactiae*) were detected in his spinal cord fluid and blood, bacterial meningitis was diagnosed, and antibiotic therapy was initiated. Although the patient’s consciousness improved, his fever persisted, and the lower limb became paler and its blood pressure had suddenly decreased. Therefore, he was transferred to our hospital. Computed tomography images showed a type B3,9 dissection according to the recent classification of TBAD, with an entry tear in the proximal descending aorta (Fig 1, *A* and *B*) and obstruction of the abdominal aorta (Fig 1, *C* and *D*).^2^ Moreover, transesophageal echocardiography had revealed a large vegetation attached to the intimal flap of the descending aorta (Fig 2, *A* and *B*). Because of the findings, we determined that emergency surgery to correct the leg ischemia and uncontrolled infection was the optimal course of action.

First, we approached the descending aorta via the left fourth intercostal space, with a retroperitoneal approach. Venous cannulation was performed from the right femoral vein, and arterial cannulation was performed from the left femoral artery. We clamped the aorta just below the subclavian artery and mid-descending artery. We used distal perfusion via partial extracorporeal circulation and monitored the blood pressure just under the distal clamp. Around the primary entry with vegetation, no other infected tissue was found, except for the vegetation. Thus, we replaced the aorta segment around the primary entry with a rifampicin-soaked prosthesis for central repair of complicated TBAD and debridement of the infected tissue. Although the patient’s lower limb blood pressure had improved compared with the preoperative value, it was approximately one half that of the patient’s upper limb blood pressure. Thus, the occluded abdominal aorta was replaced from just under the renal arteries to mid-abdominal aorta concomitantly through a median laparotomy, which resulted in sufficient lower limb blood pressure.

**Fig 3:**
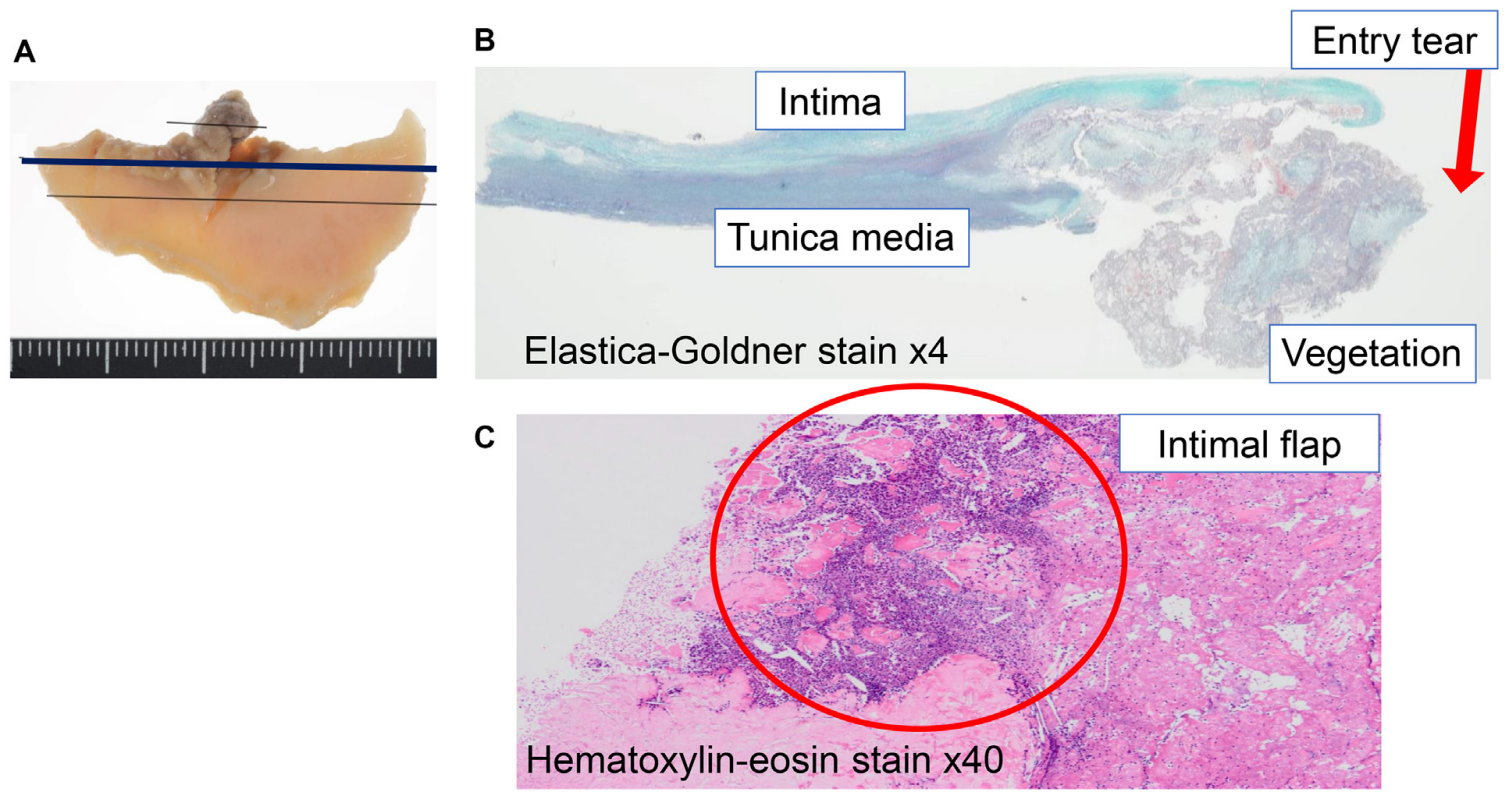
Gross and microscopic images of the entry flap. **A,** Gross view. **B,** Elastica-Goldner stain, original magnification ×4. **C,** Hematoxylin-eosin stain, original magnification ×40.

**Fig 4:**
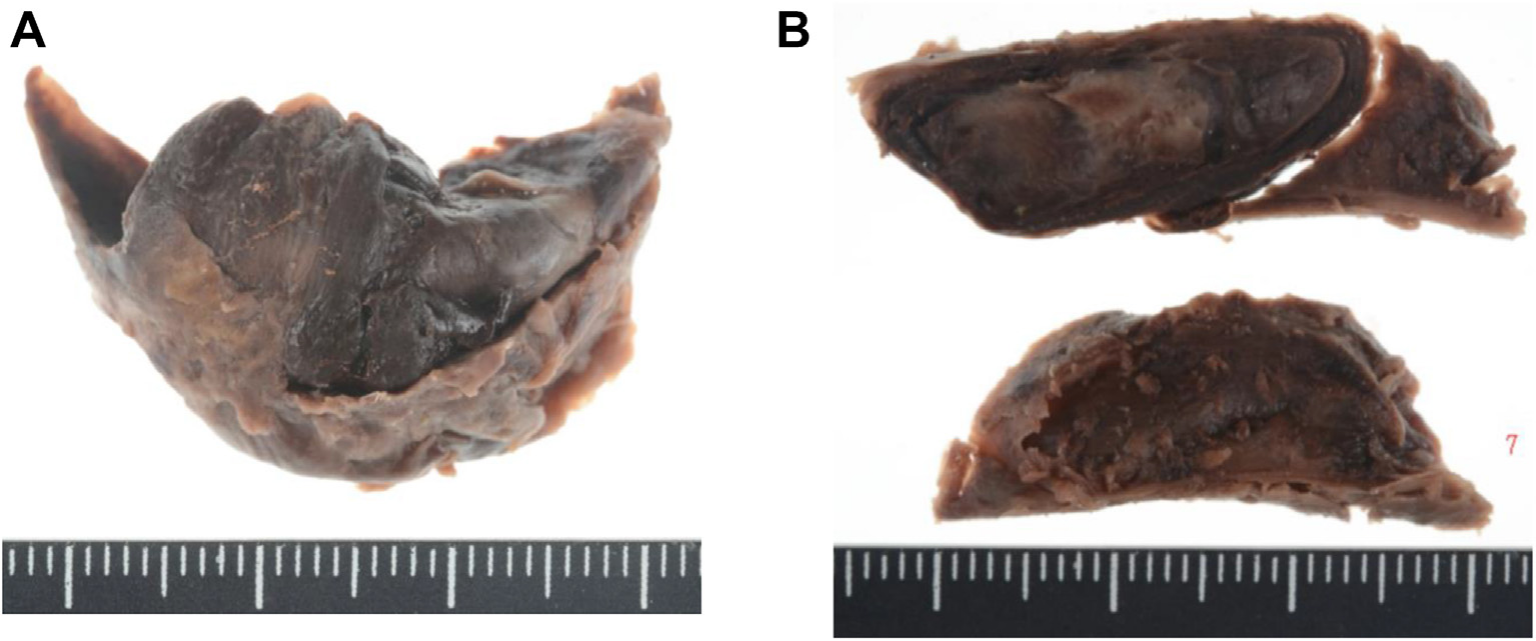
**A,** Gross image of excised abdominal aorta. **B,** Cut surfaces of thrombus.

**Fig 5:**
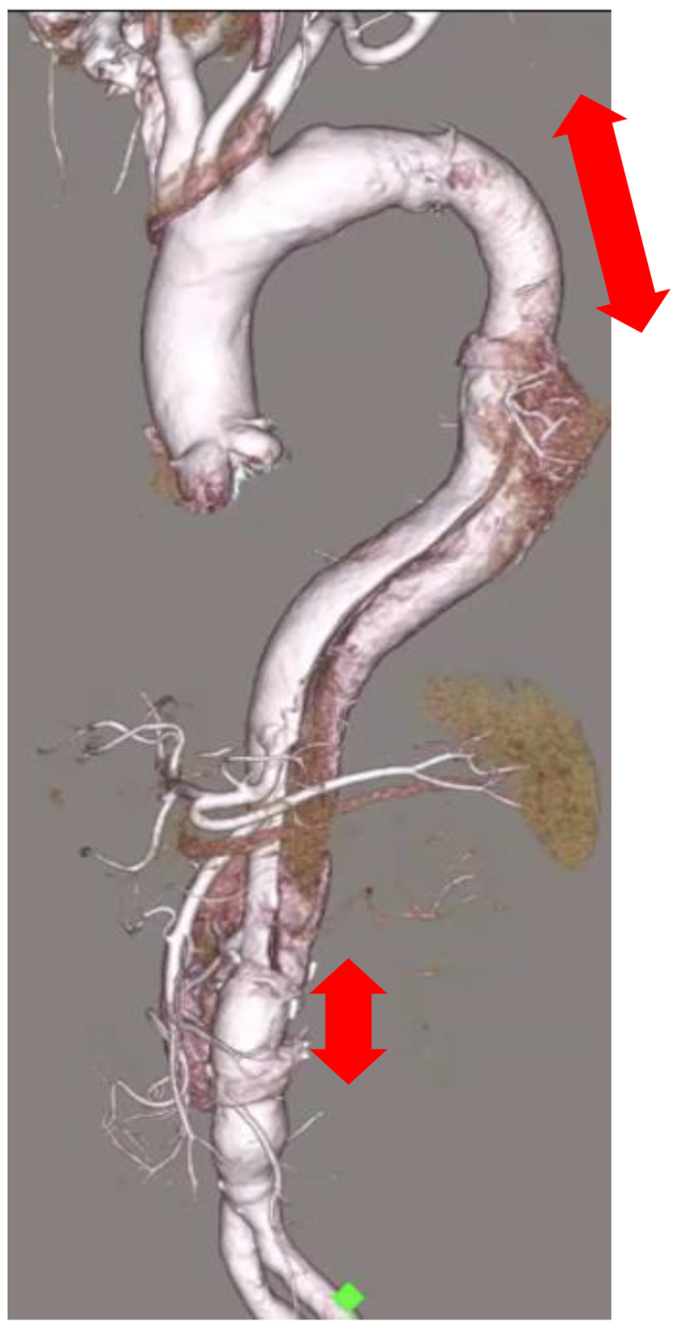
Postoperative three-dimensional computed tomography image. *Red arrows* indicate range of replacements.

The overall operation time was 6 hours, 27 minutes. The pathologic examination revealed that the vegetation had arisen from the dissection tear, the intimal flap had been infiltrated by inflammatory cells (Fig 3, *A-C*), and the excised abdominal aorta had been almost completely filled by fresh thrombus in the false lumen (Fig 4, *A* and *B*). On day 3 after surgery, the patient was moved to the general ward and was subsequently transferred to the rehabilitation facility on day 13, after confirmation of complete TBAD repair via postoperative computed tomography scan (Fig 5). After 12 weeks of antibiotics, he was discharged home and was free of recurrence of infection at the last follow-up at >7 years after treatment.

## Discussion

Mycotic vegetation in the extracardiac region is very rare.^3^ Among these cases, only a few studies have reported the occurrence of vegetation attached to the intimal flaps of the dissection.^4^^,^^5^

Birjiniuk et al^6^ demonstrated irregular hemodynamic features in an in vitro type aortic dissection model, including flow reversal, large exit tear vortices, pumping action of the mobile intimal flap, and false lumen flow reversal that had a time-averaged velocity close to stagnation. In the present case, the surgically removed intimal flap was infiltrated with inflammatory cells and was not thin and fragile, suggesting that the vegetation had developed on the proximal entry tear during the chronic phase of TBAD. Although bacterial cells were not detected in the vegetation, we considered the vegetation to be a bacterial infection, because the patient had had bacteremia preoperatively, and extensive necrotic tissue around the inflammatory cells was seen in the vegetation. The negative conversion of blood cultures postoperatively also supported this diagnosis. Because severe LLM syndrome, which will be complicated by acute TBAD in 97% of cases,^1^ had occurred during the chronic phase in the present case, the vegetation likely played a role in triggering the patient’s LLM by changing the flow pattern in the false lumen. The color Doppler echocardiogram (Fig 2; Supplementary Video) showed that the vegetation had acted as a check valve that had opened to the false lumen solely during systole. Also, the removed abdominal aorta had been almost completely filled with fresh thrombus in the false lumen, supporting this hypothesis.

During the thoracic replacement surgery we have described, we used partial extracorporeal circulation to maintain the blood flow to the patient’s abdominal organs and lower extremities. However, when the true lumen of the infrarenal abdominal aorta is severely compressed—such as occurred in our patient—evaluating the retrograde blood flow from the femoral artery using a lower limb blood pressure monitoring method can miss malperfusion of the abdominal organs. Therefore, we monitored the blood pressure just under the distal clamp site and controlled it to remain >50 mm Hg on average.

## Conclusions

We have reported a rare case of a patient with chronic TBAD complicated by LLM that had been triggered by a large vegetation on the proximal entry tear of the descending aorta. In such cases, it will be necessary to perform emergency concomitant surgical replacement of the descending and abdominal aortas carefully to prevent complications due to intraoperative organ malperfusion.

## Appendix

Supplementary Video Color Doppler echocardiogram of proximal descending aorta.
